# Trajectories of Body Height, Body Weight, BMI, and Nutrition Status from 1979 to 1987: A Measurement-Based Analysis of 8740 Montenegrin Male Adolescents from the Municipality of Berane

**DOI:** 10.3390/ijerph18105490

**Published:** 2021-05-20

**Authors:** Dusko Bjelica, Jovan Gardasevic, Zoran Milosevic, Predrag R. Bozic, Bojan Masanovic

**Affiliations:** 1 Faculty for Sport and Physical Education, University of Montenegro, 81400 Niksic, Montenegro; sportmont@t-com.me (D.B.); jovan@ucg.ac.me (J.G.); 2 Montenegrin Sports Academy, 81000 Podgorica, Montenegro; 3 Faculty of Sport and Physical Education, University of Novi Sad, 21000 Novi Sad, Serbia; zoranaisns29@gmail.com; 4 Serbian Institute of Sport and Sports Medicine, 11030 Belgrade, Serbia; predrag.bozic@rzsport.gov.rs

**Keywords:** morphological characteristics, young males, secular trend, Montenegro

## Abstract

This study aimed to consolidate the body height, body weight, BMI, and nutrition status data of the overall young male population from the Municipality of Berane in order to assess the trajectories of those variables from 1979 to 1987. The sample of respondents included 8740 adolescents who were divided into nine groups according to their age. The sample of variables included body height, body weight, body mass index, and nutrition status, which were presented based on a long-established BMI categorization (underweight, normal weight, pre-obese, and obese). The descriptive statistics are expressed as the mean and standard deviation for each variable; the analysis of nutrition status was calculated based on BMI, while LSD post hoc testing with ANOVA was employed to investigate differences between the means. The results indicate that a secular trend is visible regarding body height and body weight, while no trend is visible for the BMI and nutrition status. This study’s contribution is that it provides insight into more recently published data for the studied period and in this municipality, which can significantly aid in following the secular trend throughout Montenegro.

## 1. Introduction

Height and weight are good indications of an individual’s well-being, and deviations in weight and BMI may indicate the presence of illness or problems with nutrition [1,2,3]. However, in order to know which values are normal and which deviate, there must be growth references for all these parameters made on the basis of a large number of healthy individuals [4].

In recent decades, rapid economic development and better living standards have enabled positive secular trends in children and adolescents’ physical growth [5,6,7,8]. Due to this progressive change in mean growth values, which can be observed in most industrialized countries around the world [9,10], as well as in Montenegro [11], these parameters must be updated from time to time [2].

Other population-level changes have been present over the previous four decades. A global obesity epidemic is proven by progressive increases in both the mean BMI and in the prevalence of obesity in youngsters [12,13]. From 1975 to 2016, the rates of obesity for children and adolescents increased from 0.9% to 7.8% [14]. However, because these changes did not occur at the same time in all parts of the world, nor at the same pace [4,15], and also due to the specific physique of Montenegrins [16,17,18], new, hitherto unpublished data from any part Montenegro will be beneficial for further public health research.

Several studies dealing with the evaluation of changes in body height in Montenegrin adults exist, as well as those that monitor underweight, overweight, or obesity problems in school children [11,19,20]. It is important to note that few local studies have recently emerged that observe average body weight and BMI changes for an extended period of time [21,22,23,24], but they are limited to local samples. In order for the data to be generalized, scientists should dispose of data on the entire population, which exist in government key registers.

For this reason, the University of Montenegro contacted the Ministry of Defence and obtained permission to access data from the medical records of each young adolescent that were stored in the old archives of the Yugoslav People’s Army. The result is the present study, which merged the body height, body weight, and BMI data of all young male inhabitants of the Berane Municipality, which is the third largest city in northern Montenegro. The intent was to evaluate the trends from 1979 to 1987 for the purpose of collecting information on possible acceleration as well as the trajectories of nutrition status in children and adolescents.

## 2. Materials and Methods

The population of this retrospective cross-sectional study consists of male citizens of the younger population of Berane Municipality. They underwent medical examinations that were mandatory and conducted in order to determine their ability to perform military service. Most military candidates approached this examination before the age of 18, but military service may be postponed until the age of 27 (with a good reason, such as currently impaired health). Therefore, some of the candidates had their medical examinations later, which increased the average age in each generation. Covering the period from 22 May 1979 to 14 December 1987, 8836 candidates of the Yugoslav People’s Army with permanent residence in Berane took part in this examination, but male adolescents that were born in 1957 (6 respondents), 1958 (18 respondents), 1959 (40 respondents), 1960 (31 respondents), and 1979 (1 respondent) were excluded from the analysis because their numbers were insufficient to reliably describe an entire generation. Consequently, the analyzed data in this study include the sample of 8740 (18.12 ± 0.54 y) military candidates separated into nine groups: 585 respondents born in 1961 (18.13 ± 0.68 y), 1148 respondents born in 1962 (17.89 ± 0.44 y), 1178 respondents born in 1963 (18.46 ± 0.92 y), 630 respondents born in 1964 (17.85 ± 0.42 y), 1168 respondents born in 1965 (17.86 ± 0.37 y), 993 respondents born in 1966 (18.05 ± 0.38 y), 1073 respondents born in 1967 (18.22 ± 0.37 y), 1052 respondents born in 1968 (18.21 ± 0.22 y), and 913 respondents born in 1969 (18.38 ± 0.21 y).

Anthropometric measurements took place in a medical ambulance, and respondents were measured wearing just underwear. Body height and body weight were taken out of the sample of measurements collected for this research; using those data, the BMI was calculated. To assess body height and body weight, a standard medical scale with sliding weights with a fixed stadiometer was used. Following the basic rules and principles of the International Biological Program (IPB), the anthropometric measurement was applied, and body mass index was calculated based on the protocol handbook for physical form assessment connected to health [25]. Age-specific body mass index (BMI—kg/m^2^) cut-off points for different categories of body weight status (thinness, normal weight, overweight, and obesity) were applied according to widely used classification in the International Obesity Task Force (IOTF) [26].

Using SPSS 20.0 software (manufacturer, Chicago, IL, USA) adjusted for operation on personal computers, the data obtained during research were processed. Descriptive statistics are shown as the mean and standard deviation for all variables separately, and post hoc testing with ANOVA was used to explore differences between the means. Statistical significance was set at *p* < 0.05. The analysis of nutritional status was performed on the basis of body mass index (underweight, normal weight, pre-obese, and obese) [27].

## 3. Results

Table 1 shows the average body height, body weight, and BMI values of young male subjects from the Municipality of Berane. The average body height of all respondents covered by this study was 175.99 ± 6.78 cm. The tallest group on average consisted of respondents born in 1969 (178.90 ± 6.51), while the shorter respondents were those born in 1965 (174.79 ± 6.61). The average body weight of all respondents covered by this study was 66.81 ± 8.28 kg. The heaviest group on average also consisted of the respondents born in 1969 (68.41 ± 8.75), while the least heavy respondents were those born in 1962 (65.88 ± 7.65). The average body mass index of all respondents covered by this study was 21.55 kg/m^2^. The highest values on average were for respondents born in 1968 (21.79), while the lowest values were for those born in 1964 (21.24).

**Figure 1:**
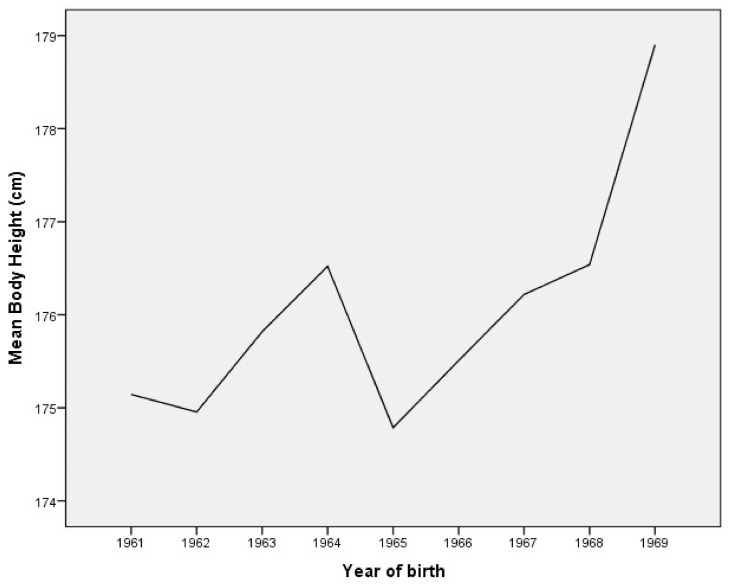
The mean body height trends by year of birth in young males.

**Figure 2:**
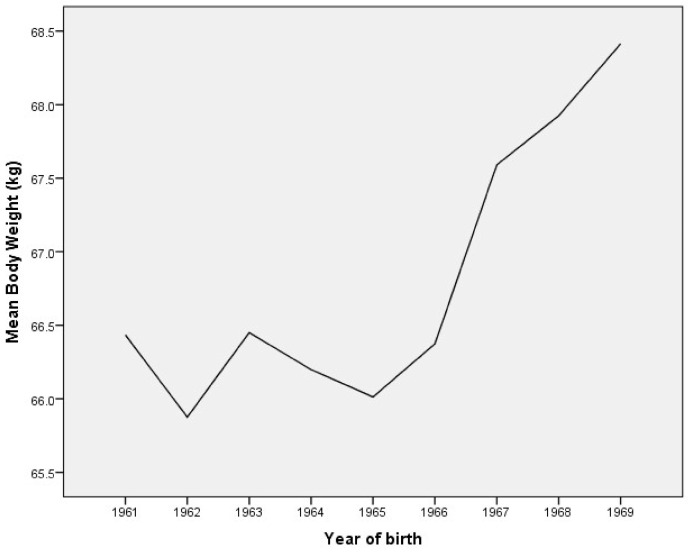
The mean body weight trends by year of birth in young males.

**Figure 3:**
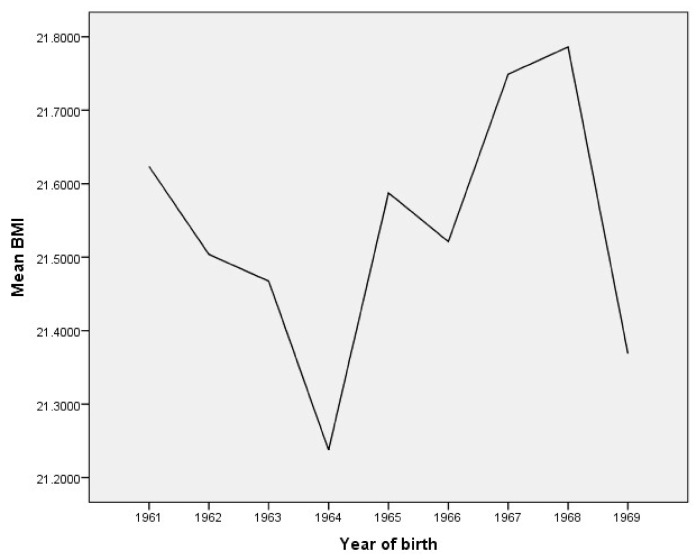
The mean body mass index (BMI) trends by year of birth in young males.

The mean body height, body weight, and body mass index (BMI) trends are presented graphically, by year of birth (Figure 1, Figure 2 and Figure 3).

Analysis of the differences in body height, body weight, and body mass index of all young male inhabitants of the Municipality of Berane is shown in Table 2, Table 3 and Table 4. LSD post hoc testing with ANOVA shows that statistically significant differences among examined groups were found.

ANOVA confirms that the mean body height of young males from the Municipality of Berane significantly increased between 1961 and 1969 (F = 33.267; *p* = 0.000). Post hoc testing revealed statistical significance for higher values of body height for subjects born in 1969 compared to all other groups (Table 2). Moreover, the subjects born in 1961, 1962, and 1965 are significantly shorter than those born between 1967 and 1969.

ANOVA confirms that the mean body weight of young males from the Municipality of Berane changed significantly between 1961 and 1969 (F = 12.365; *p* = 0.000). Post hoc testing revealed statistical significance for higher values of body weight for subjects born between 1967 and 1969 compared to all other groups, while they did not differ significantly from each other (Table 3). Furthermore, subjects born between 1961 and 1966 are evidently shorter than respondents born between 1967 and 1969; however, they also do not differ significantly from each other.

ANOVA confirms that the mean BMI of young males from the Municipality of Berane changed significantly between 1961 and 1969 (F = 5.282; *p* = 0.000). LSD post hoc testing revealed statistical significance for higher values of body mass index for subjects born in 1967 and 1968 compared to all groups of subjects, except those born in 1961 whose body mass indexes are third in rank and, of course, do not differ from each other (Table 4). In contrast, subjects born in 1962, 1963, 1964, 1966, and 1969 have significantly lower body mass indexes than subjects born in 1967 and 1968, while there is no significant difference between them.

From Table 5 it can be seen that, in all respondents covered by this study, 6.22% were underweight, 87.3% were normal weight, 6.14% were pre-obese, and 0.33% were obese. The highest percentage of subjects who were underweight is in the group of respondents born in 1969 (10.19%), while the lowest percentage is in the group of respondents born in 1963 (4.84%). When it comes to the percentage of subjects with normal body weight, the situation is reversed. The highest percentage of subjects with normal body weight is in the group of respondents born in 1963 (89.81%), while the lowest percentage is in the group of respondents born in 1969 (82.15%). The highest percentage of pre-obesity is again in the group of respondents born in 1965 (7.45%), while the lowest percentage is in the group of respondents born in 1962 (4.52%). Finally, the highest percentage of obesity is in the group of respondents born in 1968 (0.76%), while the lowest percentage is in the group of respondents born in 1966 (0.1%).

**Figure 4:**
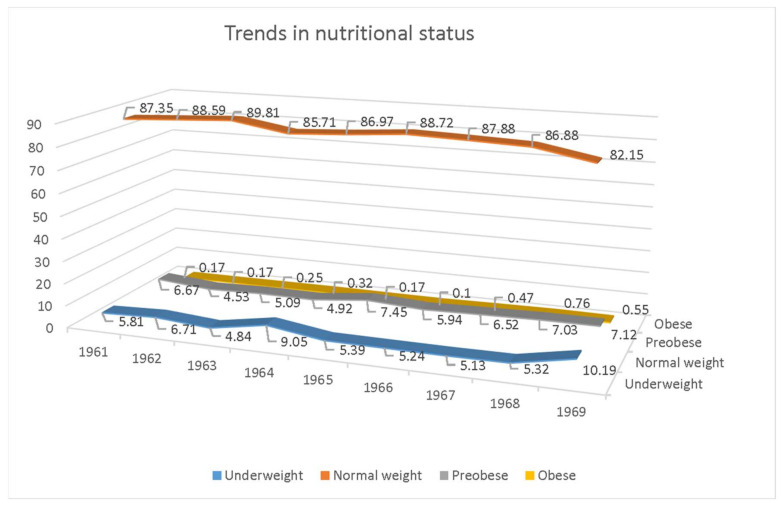
Trends in the nutrition status in young males from the Municipality of Berane.

Trends in all previously mentioned categories of nutrition status are presented graphically by the year of birth (Figure 4).

## 4. Discussion

The purpose of this study was to present new data, which were necessary to accurately monitor the rate of change in the average body height, body weight, and BMI of young Montenegrins in previous years. The results show that the mean body height of young males from the Municipality of Berane increased between 1961 and 1969 significantly (F = 33.267; *p* = 0.000), with the same case for body weight (F = 12.365; *p* = 0.000). It can be seen that respondents born between 1967 and 1969 were significantly taller and heavier than respondents born earlier. These data are not surprising because earlier studies indicate that the secular trend in body height ranged from 0.26 to 3 cm per decade in most European countries [28,29,30,31]. When it comes to body mass index, the mean values significantly differ between 1961 and 1969 (F = 5.282; *p* = 0.000), but this only indicates a large difference between subjects born in different years. There is no evidence to suggest the existence of a secular trend for body mass index. Regarding the nutrition status, the differences in categories (underweight, normal weight, pre-obese, and obese) for subjects born between 1961 and 1969 were not counted, and only a description for each group was shown. The percentage of underweight subjects was higher for the group born in 1969, the percentage of subjects with normal weight was lower for the group of subjects born in 1969, and the percentage of obesity increased for subjects born between 1967 and 1969 (which can be seen in Figure 4; however, the differences in percentages were so low that, consequently, it was concluded that the secular trend is not evident for this nine-year period.

It is interesting to remember that Montenegrins were the tallest people at the beginning of the 20th century, but later, that changed. At the beginning of the 21st century, they were pushed to second place by the Dutch [11,32,33]. It should be noted that the Dutch have grown by 14.37 cm in a little over 100 years, while the Montenegrins have grown by only 6.36 cm in the same period [11,16]. However, there are no scientific studies that could answer the question of whether the growth trend has been constant over the years or whether certain periods particularly influenced the growth and development of Montenegrins. The answer to this question has long been elusive, and the publication of this study will not reveal the exact truth. Nevertheless, this research will reduce the existing gap and enable the expansion of the database, as well as direct future research along the right path in order to reach the final answer. Montenegrin adolescents enrolled in this study (from 1979 to 1987) were, on average, 175.99 cm tall, which is 1.01 cm less than the average body height of Montenegrins recorded at the beginning of the century. Only men born in 1867 (176.22 ± 6.27) and 1968 (176.54 ± 6.83) can be said to approach the height of Montenegrins from the beginning of the 20th century, while only men born in 1969 (178.90 ± 6.51) surpass them in average height. These data lead us to the conclusion that until 1987, the secular trend in Montenegro was not noticeable, which is contrary to the results of previous studies, which showed that the mean body heights in males from 15 Western European countries underwent positive secular trends in the second half of the 20th century, ranging from 0.26 to 3 cm per decade [28,29,30,31].

However, the course of change in body height in the population was not the same over the entire period studied. It is not specified when the rate of the secular trend was at its highest, but the existence of regional differences and the impact of living conditions are emphasized. Accordingly, the reason that young males from the Municipality of Berane until 1987 have an average height below 177 cm, recorded at the beginning of the 20th century, may be because the inhabitants of this region participated in four wars between 1912 and 1945, followed by more than two decades of poor life and state building in the population in SFR Yugoslavia, of which Montenegro was an integral part; it was not until the early 1980s that every household was electrified, connected to the water supply and sewerage system, and had a bathroom and toilet [34].

Furthermore, there are no longitudinal studies that monitor the average body weight and body mass index in Montenegro; nevertheless, the results of respondents from this study can be compared with those from a study conducted by Gardasevic et al. [19]. The average value of subjects measured from 1979 to 1987 was 66.62 ± 8.35 kg for body weight and 21.55 ± 2.29 kg/m^2^ for body mass index, and the average value of Montenegrin male adolescents measured in 2015 is 79.37 kg (17 y) or 78.5 kg (18 y) for bodyweight and 24.9 kg/m^2^ (17 y) or 22.8 kg/m^2^ (18 y) for body mass index, which is significantly higher for both parameters. Even the respondents born in 1987, who have the highest value of body weight (68.41 ± 8.75), are not nearly as heavy as the respondents from 2015. These changes are in accordance with the results from 189 countries, in which the average body mass index value increased for youngsters by more than 0.05 kg/m^2^ every decade [35].

Finally, it is important to note that the percentages of subjects for categories of nutrition status (underweight, normal weight, pre-hangings, and hangings) cannot be properly compared with the current situation on the ground that similar research in Montenegro was performed only for school children (not for adolescents). However, evidence has been found in the literature that the percentage of children with normal weight is currently significantly lower when compared to the results of this study, while the percentage of subjects belonging to the pre-obese and obese groups is significantly higher [20,36,37]. Nevertheless, this is in line with the results of the NCD Risk Factor Collaboration study [35], because obesity in the previous 40 years among youngsters increased from 0.9% to 7.8% in 189 countries.

It is important to emphasize that this study has certain limitations. The first is that the subjects included in this study had an average age of 18.12 y (±0.54); most of them had not reached adulthood at the time of measurement, so it can be realistically concluded that at that moment, they had not yet completed growing [38,39]. If the measurement had been made one or two years later, their body composition data would surely have had different values. Therefore, data on their final characteristics cannot be considered completely reliable. 

The second limitation is the fact that it measured the inhabitants of only one city in Montenegro and that the results would be more objective if the sample included other cities.

Finally, the third limitation is the lack of a broader description of the socio-economic context of the population in the Municipality of Berane for the period of from 1979 to 1989. It is known that parameters such as family economic status or level of education, affect weight and growth as well as nutritional status. Only with these parameters could the state of the analyzed variables for the mentioned period be explained.

However, the strength of this study, which provides it sufficient credibility, is that it covers a complete sample of the population of the Municipality of Berane (which includes 10,954 respondents) and gives a clear picture of what the situation was at that location on this issue in the 1980s. In combination with similar studies published for Cetinje, Niksic, Bar, and Podgorica [21,22,23,24], when the data for other Montenegrin municipalities will be published, the strength of this study will be even greater, because there will be a clear picture of the situation in the entire country.

## 5. Conclusions

It can be concluded that the results obtained in this study show a visible secular trend of male adolescents in the Municipality of Berane for the period from 1979 to 1989 in terms of body height and body weight. Furthermore, this trend is not visible when it comes to BMI and assessment of their nutritional status. The results of this study give a clear picture of what the situation was at that location on this issue in the 1980s.

## Figures and Tables

**Table 1:** Descriptive data of young males from the Municipality of Berane enrolled in the study.

		Mean ± SD		
Year of Birth	Age (years)	Body Height (cm)	Body Weight (kg)	Body Mass Index (kg/m^2^)
1961 (*n* = 585)	18.13 ± 0.68	175.14 ± 6.72	66.43 ± 8.26	21.62 ± 2.12
1962 (*n* = 1148)	17.89 ± 0.44	174.95 ± 6.61	65.88 ± 7.65	21.50 ± 2.04
1963 (*n* = 1178)	18.46 ± 0.92	175.82 ± 6.79	66.45 ± 8.16	21.47 ± 2.06
1964 (*n* = 630)	17.85 ± 0.42	176.52 ± 7.17	66.20 ± 8.13	21.24 ± 2.26
1965 (*n* = 1168)	17.86 ± 0.37	174.79 ± 6.61	66.01 ± 8.20	21.59 ± 2.26
1966 (*n* = 993)	18.05 ± 0.38	175.51 ± 6.83	66.37 ± 8.37	21.52 ± 2.18
1967 (*n* = 1073)	18.22 ± 0.37	176.22 ± 6.27	67.59 ± 8.33	21.75 ± 2.28
1968 (*n* = 1052)	18.21 ± 0.22	176.54 ± 6.83	67.92 ± 8.37	21.79 ± 2.36
1969 (*n* = 913)	18.38 ± 0.21	178.90 ± 6.51	68.41 ± 8.75	21.37 ± 2.44
1961–1969 (*n* = 8740)	18.12 ± 0.54	175.99 ± 6.78	66.81 ± 8.28	21.55 ± 2.23

**Table 2:** Differences in body height values presented by year of birth.

Year of Birth	1961	1962	1963	1964	1965	1966	1967	1968
1962	0.578							
1963	0.046	0.002						
1964	0.000	0.000	0.033					
1965	0.291	0.544	0.000	0.000				
1966	0.296	0.056	0.281	0.003	0.012			
1967	0.002	0.000	0.156	0.367	0.000	0.016		
1968	0.000	0.000	0.011	0.957	0.000	0.000	0.269	
1969	0.000	0.000	0.000	0.000	0.000	0.000	0.000	0.000
			F = 33.267; *p* = 0.000					

**Table 3:** Differences in body weight values presented by year of birth.

Year of Birth	1961	1962	1963	1964	1965	1966	1967	1968
1962	0.182							
1963	0.970	0.093						
1964	0.618	0.429	0.536					
1965	0.313	0.688	0.199	0.649				
1966	0.888	0.163	0.830	0.676	0.310			
1967	0.006	0.000	0.001	0.001	0.000	0.001		
1968	0.000	0.000	0.000	0.000	0.000	0.000	0.350	
1969	0.000	0.000	0.000	0.000	0.000	0.000	0.027	0.189
			F = 12.365; *p* = 0.000					

**Table 4:** Differences in body mass index values presented by year of birth.

Year of Birth	1961	1962	1963	1964	1965	1966	1967	1968
1962	0.288							
1963	0.164	0.693						
1964	0.002	0.016	0.036					
1965	0.748	0.365	0.190	0.001				
1966	0.376	0.857	0.574	0.012	0.489			
1967	0.272	0.009	0.003	0.000	0.086	0.020		
1968	0.156	0.003	0.001	0.000	0.036	0.007	0.701	
1969	0.030	0.171	0.315	0.255	0.026	0.135	0.000	0.000
			F = 5.282; *p* = 0.000					

**Table 5:** The nutrition status by age and total for the young males from the Municipality of Berane enrolled in the study.

Year of Birth	Total	Underweight		Normal Weight		Pre-Obese		Obese	
	*n*	*n*	%	*n*	%	*n*	%	*n*	%
1961	(*n* = 585)	34	5.81	511	87.35	39	6.67	1	0.17
1962	(*n* = 1148)	77	6.71	1017	88.59	52	4.53	2	0.17
1963	(*n* = 1178)	57	4.84	1058	89.81	60	5.09	3	0.25
1964	(*n* = 630)	57	9.05	540	85.71	31	4.92	2	0.32
1965	(*n* = 1168)	63	5.39	1016	86.97	87	7.45	2	0.17
1966	(*n* = 993)	52	5.24	881	88.72	59	5.94	1	0.10
1967	(*n* = 1073)	55	5.13	943	87.88	70	6.52	5	0.47
1968	(*n* = 1052)	56	5.32	914	86.88	74	7.03	8	0.76
1969	(*n* = 913)	93	10.19	750	82.15	65	7.12	5	0.55
1961–1969	(*n* = 8740)	544	6.22	7630	87.30	537	6.14	29	0.33

## Data Availability

Not applicable.
